# Faecal Microbiota Transplantation Engraftment After Budesonide or Placebo in Patients With Active Ulcerative Colitis Using Pre-selected Donors: A Randomized Pilot Study

**DOI:** 10.1093/ecco-jcc/jjae043

**Published:** 2024-04-04

**Authors:** Emilie van Lingen, Sam Nooij, Elisabeth M Terveer, Emily Crossette, Amanda L Prince, Shakti K Bhattarai, Andrea Watson, Gianluca Galazzo, Rajita Menon, Rose L Szabady, Vanni Bucci, Jason M Norman, C Janneke van der Woude, Sander van der Marel, Hein W Verspaget, Andrea E van der Meulen-de Jong, Josbert J Keller

**Affiliations:** Department of Gastroenterology and Hepatology, Leiden University Medical Center, Leiden, The Netherlands; Department of Medical Microbiology, Leiden University Medical Center, Leiden, The Netherlands; Department of Medical Microbiology, Leiden University Medical Center, Leiden, The Netherlands; Vedanta Biosciences, Cambridge, MA, USA; Vedanta Biosciences, Cambridge, MA, USA; University of Massachusetts Chan Medical School, Department of Microbiology and Physiological Systems, Worcester, MA, USA; Vedanta Biosciences, Cambridge, MA, USA; Vedanta Biosciences, Cambridge, MA, USA; Vedanta Biosciences, Cambridge, MA, USA; Vedanta Biosciences, Cambridge, MA, USA; Ferring Pharmaceuticals, San Diego, CA, USA; University of Massachusetts Chan Medical School, Department of Microbiology and Physiological Systems, Worcester, MA, USA; Vedanta Biosciences, Cambridge, MA, USA; Department of Gastroenterology and Hepatology, Erasmus Medical Center, Rotterdam, The Netherlands; Department of Gastroenterology and Hepatology, Haaglanden Medisch Centrum, den Haag, The Netherlands; Department of Gastroenterology and Hepatology, Leiden University Medical Center, Leiden, The Netherlands; Department of Gastroenterology and Hepatology, Leiden University Medical Center, Leiden, The Netherlands; Department of Gastroenterology and Hepatology, Leiden University Medical Center, Leiden, The Netherlands; Department of Gastroenterology and Hepatology, Haaglanden Medisch Centrum, den Haag, The Netherlands

**Keywords:** ulcerative colitis, inflammatory bowel disease, faecal microbiota transplantation, Microbiome

## Abstract

**Background:**

Faecal microbiota transplantation [FMT] shows some efficacy in treating patients with ulcerative colitis [UC], although variability has been observed among donors and treatment regimens. We investigated the effect of FMT using rationally selected donors after pretreatment with budesonide or placebo in active UC.

**Methods:**

Patients ≥18 years old with mild to moderate active UC were randomly assigned to 3 weeks of budesonide [9 mg] or placebo followed by 4-weekly infusions of a donor faeces suspension. Two donors were selected based on microbiota composition, regulatory T cell induction and short-chain fatty acid production in mice. The primary endpoint was engraftment of donor microbiota after FMT. In addition, clinical efficacy was assessed.

**Results:**

In total, 24 patients were enrolled. Pretreatment with budesonide did not increase donor microbiota engraftment [*p* = 0.56] nor clinical response, and engraftment was not associated with clinical response. At week 14, 10/24 [42%] patients achieved [partial] remission. Remarkably, patients treated with FMT suspensions from one donor were associated with clinical response [80% of responders, *p* < 0.05] but had lower overall engraftment of donor microbiota. Furthermore, differences in the taxonomic composition of the donors and the engraftment of certain taxa were associated with clinical response.

**Conclusion:**

In this small study, pretreatment with budesonide did not significantly influence engraftment or clinical response after FMT. However, clinical response appeared to be donor-dependent. Response to FMT may be related to transfer of specific strains instead of overall engraftment, demonstrating the need to characterize mechanisms of actions of strains that maximize therapeutic benefit in UC.

## 1. Introduction

The inflammatory bowel disease [IBD] ulcerative colitis [UC] is a chronic disorder of the colon. The exaggerated immune response appears to be mediated by an interaction between genetic and environmental factors and the gut microbiota.^1^ Current treatment modalities are based on local immune suppression with 5-aminosalicylates [5-ASAs], temporary prednisolone/budesonide, thiopurines, or targeted therapy with small molecules and/or biologics.^2^ Despite maintenance medication, most patients suffer from periods of flares and ongoing inflammation with a significant impact on quality of life, and the 20-year cumulative colectomy rate after UC diagnosis is 14%.^3^ In addition, a subset of patients experience significant side effects from currently available medication. These observations underlie the need for new treatment modalities.

IBD is characterized by a proinflammatory cytokine microenvironment with a reduced ratio of regulatory T cells [Tregs] to proinflammatory T helper 17 cells [Th17].^4^ Tregs are a subpopulation of T cells that are able to inhibit T cell proliferation and cytokine production and thereby have a role in regulating or suppressing the immune response. Analysis of the microbiome in UC patients with active disease indicates a less diverse composition compared with healthy individuals.^5^ An imbalance of dominant species has also been observed in IBD patients, with a reduction in members of the phyla Firmicutes, Bacteroidetes, or Verrucomicrobia [specifically, *Faecalibacterium prausnitzii*, Clostridium cluster IV and XIVa, and *Akkermansia muciniphila*] and an overgrowth of facultative anaerobes from the family Enterobacteriaceae [*Escherichia coli* or *Klebsiella* species].^6,7^ Furthermore, short-chain fatty acids [SCFAs], such as butyrate, are typically reduced in patients with active IBD; these organic acids are described as important metabolites to maintain intestinal homeostasis.^8^

Faecal microbiota transplantation [FMT] is an effective treatment for recurrent *Clostridioides difficile* infections by restoring a healthy gut microbiota composition.^9^ Several studies have addressed the effects of FMT for UC, showing a response rate of around 36% for patients with various treatment regimens.^10^ Of note, efficacy may be donor-dependent^11^ and response appears to be associated with engraftment of the donor microbiota.^12^ These previous studies highlight many unanswered questions about treatment of UC patients with FMT: how to identify preferred donors, and determine which pretreatments promote the donor microbiome and response.

In this randomized controlled pilot study, we investigated the effect of pretreatment with budesonide on engraftment of FMT donor microbiota in patients with mild to moderately active UC. In addition, donor microbiota-dependent effects were compared using faecal material preparations from two rationally selected donors.

## 2. Methods

### 2.1. Study design

This was a single-centre, double-blind, randomized controlled trial in adult UC patients with active mild to moderate disease. The primary outcome was the effect of pretreatment with budesonide or placebo on the engraftment of the donor microbiota after FMT in UC patients. Secondary outcomes were safety, clinical response at week 10 [4 weeks after the last FMT] based on the partial Mayo score,^13^ and at week 14 [8 weeks after the last FMT] based on the full Mayo score [partial Mayo score + endoscopic outcomes] on FMT as an induction therapy with and without budesonide pretreatment. Additional secondary outcomes included the effects of pretreatment, donor selection, and donor microbiota engraftment on clinical response. In the absence of reliable information to estimate the effect size of the treatment on the primary endpoint in this pilot study, a sample size of 12 patients per group was chosen.^14^ Due to the COVID-19 pandemic and the following national lock-down measures implemented by the Dutch government, the inclusion of patients was temporarily halted from March 2020 to June 2020.

### 2.2. Patient selection

Patients ≥18 years old with a confirmed endoscopic diagnosis of UC with mild to moderate active disease [a full Mayo score of 4–9] were enrolled. A colonoscopy with a Mayo endoscopic sub score of 1 or 2 performed <4 weeks before study entry was required. Concomitant therapy at a stable dose for at least 12 weeks with aminosalicylates, azathioprine, 6-mercaptopurine, methotrexate, and/or anti-tumour necrosis factor [TNF]-α, was permitted.

Exclusion criteria were: patients not able to give written informed consent, pregnancy, disease limited to the rectum [<15 cm from the anal verge], recent use of antibiotics [<6 weeks] or current need for systemic antibiotics, recent use of oral corticosteroids or budesonide [<8 weeks], previous surgery specifically for UC or recent intra-abdominal surgery [<12 weeks], signs of active infectious gastroenteritis/enterocolitis, cytomegalovirus [CMV] infection, abnormal renal function (estimated glomerular filtration rate [eGFR] > 30 mL/min), pre-existing leucopenia [<2.0 mm^–3^] or thrombopenia [<90.000 mm^–3^], liver function test abnormalities [>2 ULN (upper limit of normal)], previous treatment with moe than two biologics, treatment with any investigational drug in another trial [<12 weeks of randomization], or any other significant medical illnesses that might interfere with this study.

### 2.3. Preparation and Characterization of Donor Fecal Suspensions

The donor faecal suspensions were provided by the Netherlands Donor Feces Bank [NDFB], which uses standardized procedures for donor screening, collection, preparation, and storage.^15–17^ All faecal suspensions consisted of 60 g of faeces from each individual donor, with an end volume of 10% glycerol, and stored in a −80°C freezer until the morning of the FMT. From a set of 12 eligible fecal donors, a rational selection of the 2 donors used in this study (D07 and D08) was based on microbiota community composition, ability to induce T regulatory cells (Tregs) *in vivo*, and capacity to produce short-chain fatty acids (SCFAs) *in vivo*.

#### 2.3.1 Characterization of donor stool

From 12 eligible donors, 71 stool samples were investigated by metagenomic sequencing using the Nextera XT DNA library prep kit and sequenced on the Illumina NextSeq at DNA Genotek’s CLIA certified sequencing lab [DNA Genotek Inc.a]. An average of 43 million reads was obtained for each of the 71 unique donor faecal samples [83 total metagenomes]. Faecal metagenomic reads were assigned taxonomies using the One Codex platform [Reference https://www.biorxiv.org/content/10.1101/027607v2] and a custom database containing around 150 000 reference genomes developed by Vedanta Biosciences, Inc. The One Codex platform uses a *k-mer-*based assignment algorithm where metagenomic reads are compared to a database of taxon-specific k-mers and assigned to the lowest common ancestor on the One Codex phylogenetic tree. Donors were clustered by their mean relative abundance of taxonomic families across samples, using hierarchical clustering with Euclidean distance and complete linkage. Raw data for the metagenomic sequences are available upon request.

**Figure 1.**
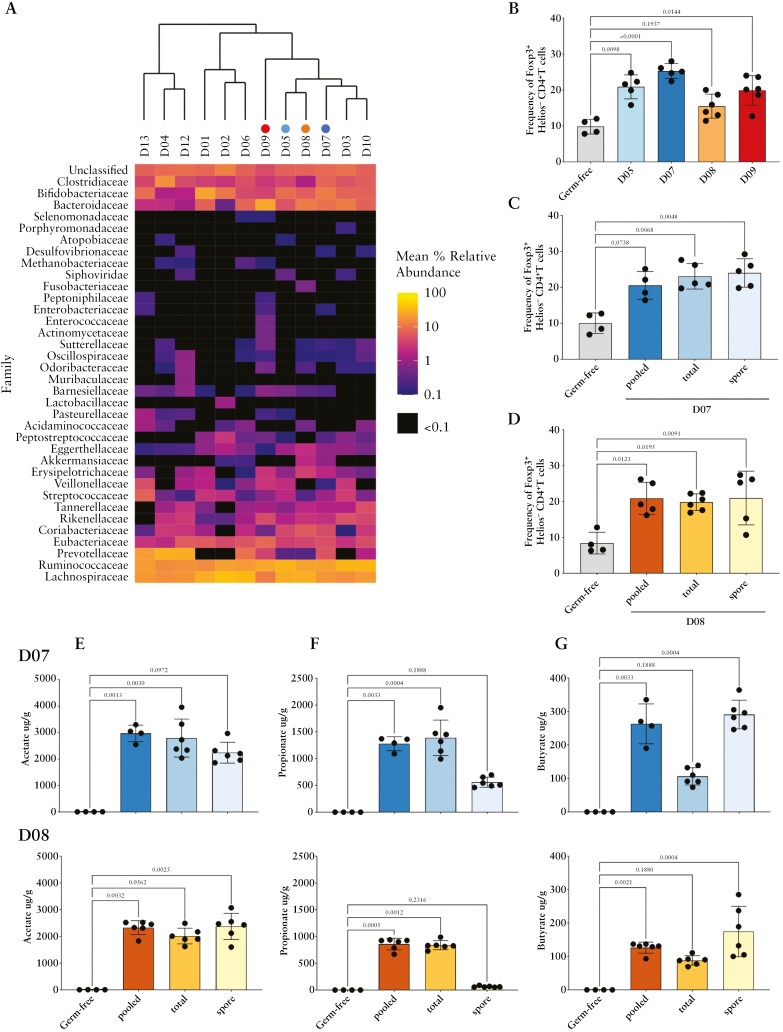
Selection of faecal microbiome transplant donors. [A] Heatmap depicting the mean abundance of taxa at the family level in stool samples from 12 healthy FMT donors. The donors are clustered based on their faecal microbiota composition. The donors selected for further evaluation based on their microbiota composition and donor availability are indicated by the filled in circles and correspond with panel B. [B–D] Donor stool induces T regulatory cells in the colonic lamina propria of mice. Germ-free mice were given either pooled [total + ethanol treated], spore-forming, or total stool fraction libraries [SFL]. Four weeks post-treatment, induced T regulatory cells in the colonic lamina propria were examined by flow cytometry [gating: lymphocytes → singlets → Live CD45+ → CD4+ CD3+ → Foxp3 vs Helios]. [B] Total SFLs from donors D09, D05, and D07 significantly induced T regulatory cells [Foxp3+ Helios−] when compared to germ-free mice; *n* = 4–6 mice per group. [C, D] Induction of T regulatory cells by pooled, total, and spore-forming SFLs from [C] D07 and [D] D08; *n* = 4–6 mice per group. [E–G] Short-chain fatty acids from caecal contents were measured by mass spectrometry. Acetate [E], propionate [F], and butyrate [G] were examined. Non-spore fractions induced higher propionate [F] while spore fractions induced higher butyrate [G]. See Supplementary appendix for detailed methods of donor selection.

Metagenomic sequencing demonstrated that all donors had high abundance of bacteria within the families Clostridiaceae, Bifidobacteriaceae, Ruminococcaceae, and Lachnospiraceae [Figure 1A]. When the donor microbiome was examined by hierarchal clustering, four donors [D13, D04, D12, and D01] clustered near each other and had high levels of Prevotellaceae, which prior studies demonstrate is detrimental in animal models of IBD.^18,19^ D02 had low levels of Prevotellaceae; however, this donor also had low levels of the propionate-producing family of bacteria Bacteroidaceae.^20^Thus, this donor was excluded from the study. A cluster of four donors [D09, D05, D08, and D07] had low levels of Prevotellaceae with high levels of Bacteroidaceae and additional taxa with potential benefit in IBD. D09 had a variety of unique taxa including additional bacterial families associated with remission in UC, such as Oscillospiraceae and Firmicutes [Peptoniphilaceae]. However, D09 did have low abundances Enterobacteriaceae and Enterococcaceae that are associated with UC.^21^ D05 had high levels of bacteria demonstrated to be beneficial in UC, and D07 and D08 had similar taxonomy to D05 but also contained Akkermansiaceae, which has been shown to provide benefit in UC.^22^ Together, the metagenomic analysis selected D05, D07, D08, and D09 for preclinical studies to examine Treg induction and SCFA production in germ-free mice.

### 2.4. Examination of Treg induction in the colonic lamina propria

For preclinical stool selection experiments, stool fraction libraries [SFLs] were generated from selected donors by culturing live bacteria and collecting plate scrapings. Spore-forming SFLs were generated by ethanol treatment of cultured live bacteria from stool. Pooled SFLs were generated by combining the spore-forming fractions with plate scrapings from total SFLs. Germ-free mice were inoculated with SFL material for 28 days prior to examining induction of Tregs in the colonic lamina propria and SCFA production in the caecum.

SFLs of total plate scrapings, ethanol-treated, and pooled [plate scrapings combined with ethanol-treated] samples were given to germ-free mice (according to the Institutional Animal Care and Use Committee [IACUC] at the Massachusetts Host-Microbiome Center Gnotobiotics Core [Boston, MA, USA]). At 28 days post-inoculation with donor SFL, colons from formerly germ-free mice and controls were collected. Mouse colonic tracts were dissected, sectioned longitudinally, cut into 2–4-mm pieces, and washed with phosphate buffered saline [PBS] to remove luminal contents. Cell suspensions from the lamina propria were prepared as described previously [^23^] with the following modifications:intestinal tissue was cut into small pieces, treated with RPMI with 10% FBS, 1 mM of DTT, and 5 mM EDTA to remove epithelial cells, and then digested with 0.2 mg/mL Collagenase VIII, and 0.075 mg/mL dispase at 37°C with shaking (180 rpm) for two 40 min periods. Cells were counted, stained with a viability dye, using the surface markers CD45, CD3, and CD4, and stained intracellularly for RORγt, Helios, Foxp3, and GATA3. Stained cells were run on a BD FACSCelesta and analysed using Flowjo and GraphPad Prism software.

When SFLs were inoculated into germ-free mice, we saw significant induction of Tregs [Foxp3+ Helios−] in mice receiving faecal material from D05, D07, and D09 [*p* < 0.02, Figure 1B]. D09 containing taxa associated with UC and D07 and D08 containing Akkermansiaceae were further assessed in germ-free experiments of non-spore, spore, and pooled SFL donor material. We found that various stool fractions from D07 and D08 were able to significantly induce Tregs [Foxp3+ Helios−] in gnotobiotic mice [*p* < 0.02, Figure 1C and D] with the exception of the pooled SFL from D07, which was not statistically significant but trended toward Treg induction [*p* < 0.08, Figure 1C].

#### 2.4.1. Short-chain fatty acid analysis

SFLs of total plate scrapings, ethanol-treated, and pooled [plate scrapings combined with ethanol-treated] samples were given to germ-free mice. At 28 days post-inoculation with donor SFL, caecal contents from formerly germ-free mice and controls were collected and mass spectrometry for SCFAs was performed [Metabolon].

Acetate was produced in germ-free mice inoculated by all SFL fractions, the non-spore, spore, and pooled SFL material, from donor microbiomes D07 and D08 [*p* < 0.1, Figure 4E]. Propionate was significantly produced by pooled and non-spore SFL fractions [*p* < 0.05, Figure 1F] as expected due to Bacteroides being the main producers of propionate. Likewise, butyrate was significantly produced by pooled and spore-forming SFL fractions [*p* < 0.005, Figure 1G] as butyrate is mainly produced by spore-forming bacteria, such as Clostridia.

Taken together, the microbial composition, Treg induction, and SCFA production of donor microbiota were used to narrow down two donor samples. Donor microbiomes were narrowed to D05, D07, D08, and D09, based on observed lower relative abundances of Prevotellaceae and abundance of the bacterial families Bacteroidaceae and Akkermansiaceae. Following observed Treg induction and SCFA production in mouse models, donors D08 and D07 were selected as FMT donors in this study.

### 2.5. Treatment schedule

**Figure 2.**
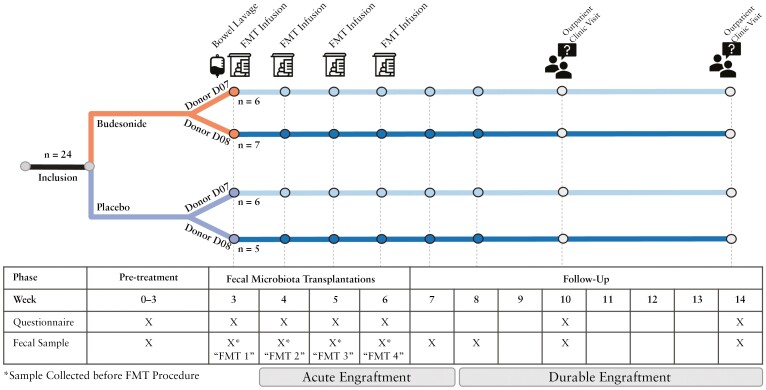
Timeline of the FECBUD study. At inclusion in the study, 24 patients were randomized into budesonide and placebo treatment groups. Patients received a bowel lavage, followed by four FMT infusions once weekly from week 3 to 6. The acute time period for assessing engraftment is defined from week 4 to 7, which corresponds to samples collected 1 week after each FMT. The durable time period for assessing engraftment is defined by samples collected 2 weeks or later following the final FMT infusion [week 8 to week 14]. In the table, X indicates when a questionnaire was used or when a sample was collected. FMT = faecal microbiome transplantation, *n* = number.

Patients were included and treated at the Department of Gastroenterology at Leiden University Medical Center [LUMC, Leiden, Netherlands]. After inclusion, patients were randomly assigned to a 3-week course of oral budesonide [9 mg] or identical looking placebo once a day [Figure 2]. The randomization procedure was conducted by an independent coordinator of the LUMC hospital pharmacy. A block diagram was used of 8 [4 to 4] patients per block for randomization, which also included stratification for donors [6 to 6]. The pretreatment phase was followed by four weekly infusions [weeks 3–6] of a donor faecal suspension from either donor D07 or D08 produced by the NDFB as described above. The donor faecal samples were infused in the duodenum via either a nasoduodenal tube or directly via the gastroscope, both according to the LUMC protocol. One day before the first FMT, patients underwent bowel lavage with 2 L of a macrogol solution [Kleanprep]. Prior to every FMT, all patients were sober for at least 6 h. During the study, no changes were allowed regarding the patients’ medication or diet.

### 2.6. Data collection

Baseline characteristics included age, sex, weight, disease duration, location of disease, previous medication, and concomitant drug treatment. A full Mayo score, both clinical and endoscopic, was assessed, and all patients underwent physical examination [weight, temperature, and pulse]. The project team [AM, SM, JK] reviewed the endoscopic Mayo score at inclusion. Analysis of blood samples included C-reactive protein, leukocytes, thrombocytes, renal function [eGFR, creatinine], liver function tests and faecal calprotectin [FCP]. To assess quality of life, the EQ-5D-5L and a disease-specific questionnaire were used.^24^ During follow-up, disease activity and adverse events were registered in a structured way. At every study visit, stool and blood samples were collected for research purposes. The questionnaires were administered again at weeks 10 and 14. At the end of the study, a sigmoidoscopy was performed to assess the endoscopic Mayo score [Figure 2].

### 2.7. Patient outcomes and clinical response

At week 10 [4 weeks after the last FMT], clinical outcome was based on the partial Mayo score, including stool frequency in 24 h [score 0–3], the presence of blood [score 0–3] and the Physician Global Assessment [score 0–3]. At week 10, remission was defined as no complaints [partial Mayo score of ≤2, with no individual sub score of >2], and partial response was defined as a decrease of at least 3 points in the partial Mayo score; all other situations were considered as no response.

At week 14 [8 weeks after the last FMT], clinical outcome was based on the full Mayo score. All endoscopic pictures were reviewed blinded by two experienced endoscopists [AM and SM]. In case of disagreement, a third reviewer [JK] scored. At week 14, remission was defined as no complaints [partial Mayo score of ≤2, with no individual sub score of >2] and an endoscopic Mayo score 0–1a [erythema without friability]. Partial remission was defined as a decrease of at least 3 points in the partial Mayo score and a decrease of at least 1 point in the endoscopic Mayo score [e.g. also a decrease from 1b to 1a]. No response at week 14 was defined as an occurrence or recurrence of symptoms and an equal or increased endoscopic Mayo score. Patients who did not complete the study because of progressive symptoms/disease were considered treatment failures [no response].

### 2.8. Metagenomic sequencing of faecal samples

Stool samples were collected according to standardized procedures^15,17^ at inclusion, before placebo or budesonide treatment, and prior to faecal microbiome transplantations and following treatment on weeks 7, 8, 10, and 14 [Figure 2]. Total DNA was extracted from donor and recipient stool samples using the DNeasy PowerSoil Pro kit [Qiagen Cat. No. 47016] on the QIAcube and the MO BIO MagAttract PowerMicrobiome [+ClearMag] kit [Cat. No. 27500-40EP] automated on the KingFisher Flex [ThermoFisher]. Metagenomic sequencing was performed by Diversigen using the Illumina NovaSeq 6000 platform [Illumina Inc.]. Samples were sequenced using the 1 × 100 bp single end protocol to a median depth of 2.9 million reads. Raw data from metagenome sequencing are available upon request.

### 2.9. Engraftment analysis

Raw metagenomic reads were uploaded to the One Codex analysis platform [One Codex]for taxonomic classification, which assigns taxonomy based on a custom, curated databased as described previously in ‘Characterization of donor stool’ above. In short, the raw reads are divided into k-mers sets then assigned taxonomy comparing the k-mers to a custom in-house reference database of microbes. Taxonomic profiling with this approach has been used previously to assess the impact of live biological therapeutics on healthy volunteer microbiomes,^25^ evaluate host microbiome response to sterile faecal filtrate transplantation in patients with C. difficile infection,^26^ and classify gut microbiome composition in patients with bloodstream infections.^27^ The taxonomic assignment table was then imported into R to calculate the engraftment score, visualize the results, and perform statistical tests [R v.4.0.2].

After applying a 0.1% relative abundance threshold, the median cumulative abundance per sample was 97%, i.e. only 3% of data were discarded. Next, we determined which species were putatively donor-derived by determining the donor core species. Species were considered ‘core’ if they were present above the detection limit in at least six samples [out of 13 or 14 for donor D07 and D08, respectively]. This yielded core microbiomes of 160 and 115 species for donor D07 and D08, respectively. These species were also abundant in all donor samples: their total abundance was always >70%. To determine which of these species engrafted in the recipient patients, these putatively donor-derived species were compared to the bacterial profiles in the recipients before FMT and overlapping species were excluded, i.e. species already present in the patient before FMT were deemed not engrafted. The species that were unique to the donor for each subject were used to quantify engraftment. The resulting median number of putatively donor-derived species per recipient was 82 [range: 62–135]. Engraftment metrics were calculated for the complete duration of the experiment [‘overall’], for the phase directly following the FMTs [Weeks 4–7, Figure 2, ‘acute’] and for the weeks after the FMTs [Weeks 8–14, Figure 2, ‘durable’]. Three different engraftment measures were then summed to produce a total engraftment score. [1] The median Species Engraftment Index [SEI], which is the median total relative abundance of the engrafted species. This quantifies the extent of displacement of the recipient microbiome with the donor microbiome. [2] The second metric is the Species Engraftment Fraction [SEF], which is the fraction of bacteria that engrafted divided by all putatively engrafted, donor-derived species [and is therefore a number between 0 and 1]. This metric describes the diversity of donor species, regardless of abundance, that are introduced to the recipient in FMT, which was previously associated with donor efficacy and clinical response.^28–30^ [3] Engraftment was assessed by comparing bacterial species compositions using pairwise Aitchison distances. Recipient to donor distances were evaluated as median distance to the corresponding donor microbiomes and normalized by the distance to the donor at the timepoint before the first FMT [after pretreatment and bowel lavage; timepoint ‘week 3’]. Next, we converted this to similarity scores by taking [1/Aitchison distance] for ease of interpretation [high score = more engraftment]. Microbiota similarity has also been used to study FMT efficacy.^12,31^ To combine the three different metrics for engraftment in a total engraftment score, we normalized the SEI, SEF, and median Aitchison distance to donor by multiplying the SEI and Aitchison distance so that each had an approximately equal median, and then summed them. The scripts used to quantify engraftment are available online at https://doi.org/10.5281/zenodo.8158623.

### 2.8. Machine learning modelling to identify microbiota signatures of response

To determine recipient microbiome taxonomic signatures that are strongly predictive of response, random forest classification [RFC] modelling was used as in the study from Haran and colleagues.^32^ Fewer microbiome samples were available at later timepoints for training and testing models because treatment failures dropped out of the study over time. This created a trade-off between maximizing the number of patients included in a model or maximizing the breadth of timepoints, which was accounted for by creating four different models. Model 1 predicted response using only baseline microbiome composition of subjects [number of subjects included, *n* = 24]. Model 2 included baseline and post-FMT microbiome samples which resulted in the exclusion of two subjects [*n* = 22]. Model 3 included baseline, post-FMT, and week 7 and week 8 samples [*n* = 18], and model 4 included all timepoints up to week 14 [*n* = 16]. Each model was run using aggregated microbial abundances at different taxonomic levels [e.g. species, genus, class, order]. To focus on highly predictive features, the RFC models were wrapped within the Boruta feature selection algorithm.^33^ The different models were compared in terms of predictive ability by estimating area undre the curve [AUC] and F1 score from a leave-one-out cross-validation [LOOCV] scheme. The most predictive model was run on the entire dataset to identify important taxa associated with response via permutated importance calculations.^34^ Finally, to derive human interpretable rules on the bacterial abundance of the different taxa that best discriminates between responders and non-responders, we passed the RFC modelling results to the Stable and Interpretable Rule Set [SIRUS] pipeline.^35^

### 2.9. Statistical analysis

Continuous variables are presented as mean with standard deviation [SD] or as median with interquartile range [IQR] depending on the normality of the underlying distribution. Baseline characteristics were compared using an independent sample t-test or Mann–Whitney U-test, and paired variables were compared using a paired sample t-test or Wilcoxon signed-rank test. Categorical variables are presented as a total percentage and compared by using the chi-square test or Fisher’s exact test. A *p*-value of ≤0.05 was considered significant for all tests. All statistical analyses other than those on engraftment scores were performed using SPSS v.25.0.

### 2.10. Ethical consideration

This research project was reviewed and approved by the Medical Ethical Committee in the LUMC, with reference number NL 65976.098.18. Informed consent was obtained from all participants prior to inclusion in the study. The study was conducted in accordance with the principles of the Declaration of Helsinki. The study was registered in the Netherlands Trial Register, with reference number NL9858.

## 3. Results

From May 7, 2019 to October 27, 2020, 24 patients were enrolled and treated. The study timeline is summarized in Figure 2. The median age of enrolled patients was 42 years [33.0–57.5] and 50% of patients were male. The median UC disease duration was 13.5 years [5.5–20.3]. The baseline median full Mayo score was 7 [5–9] with a median faecal calprotectin level of 944 µg/g [369–1719]. Although more patients in the budesonide pretreatment group had used anti-TNF therapy in the past [eight vs two; *p* < 0.05] this was not observed with respect to concomitant treatment in the study [Table 1].

**Table 1.** Patient characteristics

	Total group [*n* = 24]	Pre-treatment			Donor		
		Placebo [*n* = 11]	Budesonide [*n* = 13]	*p-*value	D07 [*n* = 12]	D08 [*n* = 12]	*p-*value
Age [years], median [IQR]	42 [33–57.5]	52 [37–73]	34 [32–49.5]	0.077	42.5 [33.3–57.5]	40.5 [31.3–61.0]	0.583
Male sex, *n* [%]	12 [50]	5 [45.5]	7 [53.8]	0.682	5 [41.7]	7 [58.3]	0.414
Disease duration [years], median [IQR]	13.5 [5.5–20.3]	15 [4–22]	13 [6–19.5]	0.663	11.0 [3.2–15.0]	15.0 [7.3–21.8]	0.214
Clinical Mayo score, median [IQR]	5.0 [4.0–7.0]	5.0 [4.0–7.0]	5.0 [4.0–7.0]	0.878	6.5 [4.0–7.0]	5.0 [4.0–7.0]	0.284
Endoscopic Mayo score, *n* [%]				1.000			1.000
Mayo 1A	0	0	0		0	0	
Mayo 1B	2 [8.3]	1 [9.1]	1 [7.7]		1 [8.3]	1 [8.3]	
Mayo 2	22 [91.7]	10 [90.9]	12 [92.3]		11 [91.7]	11 [91.7]	
Full Mayo score, median [IQR]	7.0 [5.0–9.0]	7.0 [5.0–9.0]	7.00 [6.0–9.0]	0.975	8.0 [6–9]	7.0 [5.0–9.0]	0.385
Prior anti-TNF therapy, *n* [%]	10 [41.7]	2 [18.2]	8 [61.5]	**0.047**	5 [41.7]	5 [41.7]	1.000
Concomitant drugs, *n* [%]							
5-ASA	16 [66.7]	8 [72.7]	8 [61.5]	0.679	8 [66.7]	8 [66.7]	1.000
Immunomodulators	3 [12.5]	2 [18.2]	1 [7.7]	0.576	1 [8.3]	2 [16.7]	1.000
Anti-TNF-α	3 [12.5]	0 [0.0]	3 [23.1]	0.223	2 [16.7]	1 [8.3]	1.000
Hb concentration, g/L, median [IQR]	8.3 [7.6–9.0]	8.5 [7.7–9.1]	8.1 [7.5–8.6]	0.223	8.2 [7.4–9.0]	8.3 [8.0–9.1]	0.506
White cell count, ×10^9^/L, median [IQR]	7.0 [5.5–8.2]	7.1 [4.6–7.4]	6.7 [5.5–9.7]	0.469	7.2 [4.8–8.1]	6.9 [5.5–8.4]	0.840
CRP, mg/L, median [IQR]	1.9 [1.4–5.4]	2.5 [1.4–5.5]	1.8 [1.4–5.2]	0.450	1.9 [1.4–5.8]	2.2 [1.4–5.2]	0.772
FCP, µg/g, median [IQR]	944.0 [369.0–1719.0]	1042.5 [702.8–1814.3]	841.0 [241.0–1610.5]	0.193	989.5 [452.5–1399.8]	795.0 [326.0–1976.0]	0.926

Anti-TNF = anti-tumour necrosis factor, CRP = C-reactive protein, FCP = faecal calprotectin, Hb = haemoglobin, *n* = number.

Bold indicates *p*<0.05.

### 3.1. Total engraftment of donor microbiome

To assess engraftment, one total engraftment score was computed from the weighted sum of three engraftment metrics: SEI, SEF, and Aitchison similarity of recipient microbiome to that of the donor following FMT. The relationship between each metric and donor, pretreatment, week 10 response, and week 14 response are reported in Supplementary Figures 1–4. Engraftment was calculated during FMT treatment [Acute Engraftment, Weeks 4–7; indicated in Figure 2], following treatment [Durable Engraftment, Weeks 8–14], and over the entire study [Overall Engraftment].

**Figure 3.**
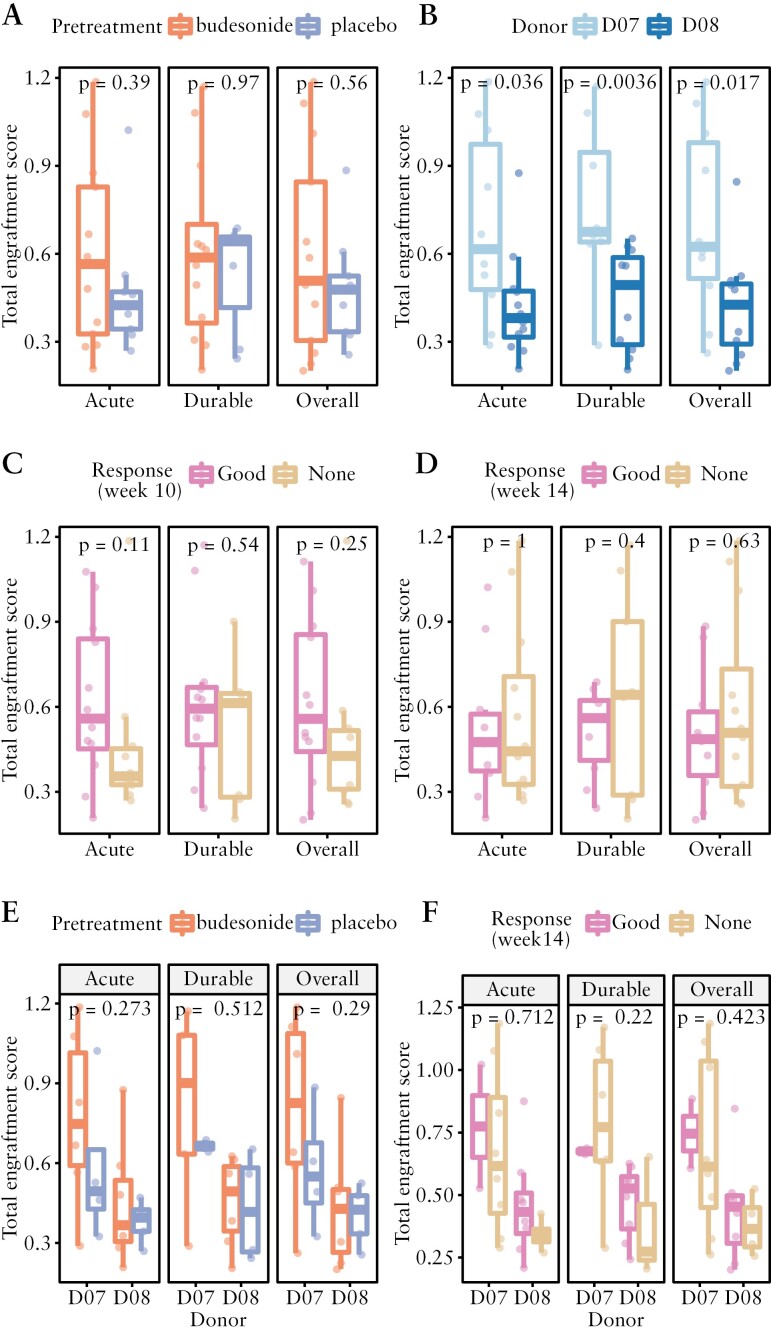
Total engraftment of donor microbiome. To test for an association between bacterial engraftment and pretreatment, donor, or clinical response, we first calculated a single engraftment score, combining metrics using species abundance, number of species, and species composition. We then used the weighted sum of these three metrics as the total engraftment score. [A] Impact of pretreatment on engraftment, [B] impact of donor, [C] association with response at week 10, [D] association with response at week 14, [E] impact of pretreatment while controlling for donor, and [F] association of response at week 14 with engraftment while controlling for donor. Donor D07 is correlated with higher acute, durable, and overall engraftment [*p* < 0.05]. All other associations were not significant [*p* > 0.05]. Panels A–D show *p*-values from Wilcoxon rank sum exact tests, while panels E and F show *p*-values from two-way ANOVA; panel E shows values dependent on the pretreatment variable [*p*-values for donor variable: Acute *p* = 0.022; Durable *p* = 0.011; Overall *p* = 0.016], and panel F shows values dependent on response [*p*-values for donor variable: Acute *p* = 0.018; Durable *p* = 0.0201; Overall *p* = 0.024]. Acute = 1 week after each FMT; Durable = 2–8 weeks after final FMT; Overall = from 1 week after the first FMT up to 2 months after FMT.

First, budesonide pretreatment was not associated with a higher total engraftment score [*p* > 0.5, Figure 3A], as compared with placebo. Further, none of the individual engraftment metrics showed a difference in engraftment between patients pretreated with budesonide or placebo [Supplementary Figure 1].

Second, we compared the total combined engraftment scores for patients who received FMT from donor D07 to those with FMT from donor D08 [Figure 3B]. Donor D07 was associated with higher total engraftment at all studied time frames [*p* < 0.04], which was driven by a higher SEF [Supplementary Figure 2B] and higher Aitchison similarity [Supplementary Figure 2C]. The median SEI was not different between donors D07 and D08 [*p* > 0.05; Supplementary Figure 2A]. To ensure the budesonide impact on engraftment was not trumped by the donor difference, two-way ANOVAs were performed on acute, durable, and overall engraftment outcomes taking into account both pretreatment and donor assignment. The effect of budesonide pretreatment on engraftment remained insignificant in the acute, durable, and overall time windows [*p* = 0.3, *p* = 0.5, *p* = 0.3, respectively] while the effect of donor remained significant given the patient’s pretreatment status [Acute *p* = 0.02, Durable *p* = 0.01, Overall *p* = 0.01, respectively, Figure 3E].

### 3.2. Clinical outcomes

The partial Mayo score did decrease from 5.69 [1.0] at baseline to 4.38 [1.3] after 3 weeks of pretreatment with budesonide, and in the placebo group the partial Mayo score did decrease from 5.64 [1.2] to 4.82 [1.7] after 3 weeks [no significant difference between groups].

At week 10, 42% [10/24] achieved clinical remission, 8% [2/24] achieved partial remission, and 50% of the patients [12/24] had no clinical response or progressive symptoms. Pretreatment with budesonide was associated with a non-significant higher response rate compared to placebo [8/12 vs 4/12 patients, *p* = 0.5] and donor randomization [*p* = 0.8] did not influence the clinical Mayo score at week 10 [Table 2].

**Table 2.** Overview of clinical outcomes and the effect of pretreatment and donors in the [partial] responder group

	Week 10	*p*-value	Week 14	*p*-value
Clinical outcomes [*n*]				
No response	50% [12/24]		58% [14/24]	
Partial remission	8% [2/24]		4% [1/24]	
Clinical remission	42% [10/24]		38% [9/24]	
[Partial] remission^*^	*N* = 12		*N* = 10	
Pre-treatment [*n*]		0.508		1.0
Placebo	4/12		4/10	
Budesonide	8/12		6/10	
Donors [*n*]		0.828		**<0.04**
D07	5/12		2/10	
D08	7/12		8/10	

^*^The effect of pretreatment/donors in the [partial] responder group.

*N* = number of patients.

At week 14, 38% [9/24] of patients achieved combined clinical and endoscopic remission, 4% [1/24] achieved partial remission, and 58% [14/24] had no response. Of the ten FMT responders at week 14 [partial remission or remission], four patients were pretreated with placebo versus patients with budesonide [*p* = 1.0]. Of note, at week 14, there was a donor-dependent effect, with 80% [8/10] of the responders receiving donor material from D08 compared with 20% [2/10] receiving donor material from D07 [*p* < 0.05, Table 2]. The 14 non-responders were treated with budesonide [*n* = 2], prednisolone [*n* = 4], anti-TNF-α [*n* = 2], or chose themselves not to start a new induction therapy [*n* = 3]. Three patients were admitted to the hospital due to worsening of their complaints, and responded well on intravenous prednisolone and anti-TNF-α.

### 3.3. Quality of life

In the responder group [*n* = 10], the median score of the EQ-5D-5L increased significantly at the end of the study compared to baseline, independent of pretreatment [see Supplementary results]. However, assessment of changes in quality of life in non-responders was unfortunately not possible, as some of them did not complete study follow-up due to progressive symptoms.

### 3.4. Adverse events

In total, 24 adverse events were observed [Table 3]. Of these, 16 events were listed as possibly related to FMT, such as abdominal cramps after infusion and constipation. Eight events were registered as not related to FMT, including: high blood pressure and high glucose level that were considered incidental findings and were treated by the general practitioner, weight gain that was attributed to cessation of smoking at the same time, stasis of food in the stomach resulting in the postponement of FMT by 1 week, and pain in a foot which was treated with paracetamol. Two cases of anaemia were attributed to ongoing inflammation and were treated with iron supplementation. None of the adverse events mentioned in Table 3 led to hospitalization.

**Table 3.** Overview of adverse events^*^.

Related to FMT [*n* = 16]	*N*	Not related to FMT [*n* = 8]	*N*
Borborygmus	2	High blood pressure	1
Abdominal cramps	5	Increasing weight	1
Increase of stool frequency	2	High glucose level	1
Vomited after infusion	2	Stasis of food in stomach	1
Constipation	3	Flu-like complaints	1
Nausea	2	Anaemia	2
		Stinging pain in foot	1

FMT = faecal microbiota transplantation, *N* = number.

^*^None of the adverse events resulted in hospitalization.

### 3.5. Influence of total engraftment on clinical response

Clinical response was not associated with higher total engraftment. Total engraftment did not differ between responders and non-responders at week 10 or week 14 [Figure 3C and D; all *p* > 0.05], although there was a trend towards more engraftment in responders at week 10 [*p* = 0.25]. Separate engraftment metrics were also not found to differ between patients with a good clinical response and those with no response [Supplementary Figures 3 and 4]. To test whether the association between engraftment and response might be donor-dependent, a two-way ANOVA was used [Figure 3F]. This test yielded no significant correlation between response and engraftment [Acute, Durable, and Overall all *p* > 0,2], but only confirmed the significant donor-effect [Acute, Durable, and Overall *p* < 0.02].

### 3.6. Taxonomic associations with FMT response

RFC modelling was used to predict the binary response versus non-response for each patient as a function of the bacterial relative abundance at different time points and aggregated at different taxonomic levels [Supplementary results]. The models considering just baseline samples [Model 1], or samples collected at baseline and during the FMT administration [Model 2], were not found to be predictive of response [Supplementary Figure 5A]. We selected the two models with higher classification accuracy [i.e. Model 3 and Model 4 both at genus-level aggregation] [Supplementary Figure 5A] to inspect microbiome signatures that are predictive of response. Model 3 considers time points up to week 8, and Model 4 considers time points up to week 14. In both models, the abundance of several genera belonging to Clostridia clusters IV and XIVa [e.g. *Roseburia*, *Dorea*] or Bacteroidales [e.g. *Bacteroides*, *Coprobacter*] post-FMT [Week 8–10, 2–4 weeks after the final FMT] or during FMT dosing [e.g. *Roseburia* FMT 2] were highly predictive of response [Supplementary Figures 5B and C]. Similarly, both models also found that the abundance of *Dialister* immediately following FMT to be discriminatory. In addition, for Model 4, five of the nine highly prognostic predictors of response involved enrichment in abundance of Clostridia members [Eubacteriales: *Agathobaculum*, *Lawsonibacter*, *Roseburia*, *Tyzzerella*, *Phascolarctobacterium*] at weeks 7–10 [1–4 weeks after the final FMT, Supplementary Figure 5B]. Similarly, Model 4 found the enrichment in Coriobacteria representatives [e.g. *Enorma*] at weeks 10–14 to be highly discriminatory between responders and non-responders. SIRUS analysis to gather human-interpretable rules for both models found the enrichment in *Dialister* immediately following FMT to be strongly predictive of no response [Supplementary Figure 6]. Conversely higher colonization by *Roseburia* [for both models], *Coprobacter* [for Model 3, Supplemental Figures 5B and 6A] or *Phascolarctobacterium* [Model 4, Supplemental Figures 5C and 6B] to be strongly indicative of response.

### 3.7. Microbiota community differences among FMT donors

**Figure 4.**
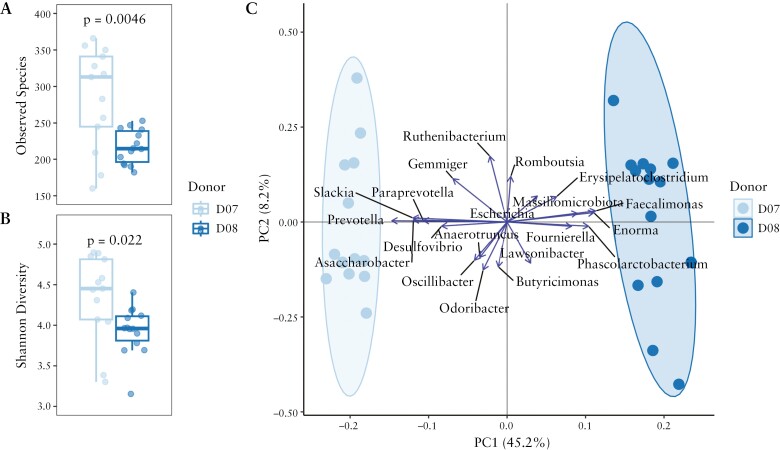
Microbiota community differences among FECBUD FMT donors. Differences in microbial diversity between D07 and D08 samples as number of observed species [A] and Shannon index [B] demonstrate that D07 has higher diversity than D08 [*p* < 0.03]. Thirteen donor samples from D07 and 14 samples from D08 were collected between June 2017 and July 2019. [A] Microbiota community differences between D07 and D08 samples as clusters [C] were significantly different by PERMANOVA [*p* = 0.001].

Although donors D07 and D08 were both found to contain high abundances of bacterial families [i.e. Bacteroideceae and Akkermansiaceae] with presumed clinical benefit to UC patients and low in presumed antagonistic bacterial families [i.e. Prevotellaceae and Enterococcaceae, Figure 1], the faecal microbiome of the two rationally selected donors were distinct [Figure 4]. Specifically, the donors differed significantly from each other in terms of richness, measured as number of observed species [D07 = 280; D08 = 220; *p* < 0.005, Figure 4A] and as evenness measured as Shannon Diversity [D07 = 4.3; D08 = 3.89; *p* < 0.03, Figure 4B]. Moreover, stool samples clustered by donor, with the overall community structure showing statistically significant differences [PERMANOVA *p* = 0.001, Figure 4C]. The D07 stool microbiome samples had greater *Prevotella*, *Slackia*, *Paraprevotella*, *Asaccharobacteria*, and *Desulfovibrio*. Collectively, these five genera comprised 0.9–9% of the total microbial composition of samples originating from D07. These genera were largely absent in D08 samples with only *Desulfovibrio* detected in five samples at <0.06% abundance. In contrast, the D08 microbiome samples contained *Enorma*, *Faecalimonas*, *Erysipelatoclostridium*, *Phascolarctobacterium*, *Fournierella*, and *Massiliomicroiota* [Figure 4C]. Collectively, these six genera comprised 0.4–1.3% of the overall microbial composition with a median total relative abundance of the six genera collectively of 0.6%. *Erysipelatoclostridium* was the only genus of the six detected in any D07 samples, where it was detected in one sample at 0.04% abundance.

## 4. Discussion

In several randomized controlled trials, microbiota transplantation has been described as a promising induction treatment strategy for patients with UC.^10^ However, the optimal selection of donors and patients, and the correlation between engraftment of donor microbes and clinical outcomes is not yet known. In this small, randomized study, overall response or remission after FMT treatment was 42%, which appears similar to previous studies.^10,11^ After budesonide pretreatment, the engraftment of donor microbiota and clinical response appeared slightly higher, but these effects were not statistically significant. Also, total engraftment did not predict treatment response significantly. However, our study confirmed the previous observation of a donor-dependent effect of FMT in patients with UC.

A number of studies suggest that FMT success is dependent on the microbial diversity and composition of the stool donor, leading to the proposition of the existence of FMT super-donors.^36^ In our study, two donors were rationally selected based on their microbiota profile, the ability to induce Tregs, and the capacity to produce SCFAs *in vivo.* The selected donors had high abundance of bacteria within the families Clostridiaceae, Bifidobacteriaceae, Ruminococcaceae, and Lachnospiraceae, all known as important components of the gut commensal bacteria in healthy people,^7^ and Akkermansiaceae, which has been shown to provide additional benefit in UC.^22^ In addition, the selected donors had low abundance of Prevotellaceae compared to all other donors considered for the study, as in IBD mouse models colonization with Prevotellaceae seems to be detrimental.^19^ Surprisingly, we observed a significant difference in efficacy between those two donors, in favour of donor D08 that showed lower Treg induction in germ-free mice compared to D07. FMT preparations from this donor induced clinical remission in 80% of patients. Similarly, Moayyedi *et al*. found one donor with significantly higher response rates than other donors, with a remission rate of 39% in recipients who received FMT preparations of the successful donor.^11^ Of note, the total number of species and the species diversity in our clinically more effective donor were lower. This agrees with recently published observations by Podlesny *et al*.^37^ and thereby questions whether microbiome diversity of donors predicts clinical outcome, as suggested previously.^29,30^ These findings also suggest that selecting FMT donors based on *in vitro*, microbiome community, or animal model data alone may be insufficient to predict FMT clinical response in patients with IBD. Therefore, more research isolating and identifying bacterial strains responsible for mediating response is required to provide more consistent results for microbiome-mediated therapies in UC patients.

The engraftment of the donor microbiota can be predicted from the abundance of bacteria in the donor and the pre-FMT patient,^37,38^ and is thereby considered as an informative endpoint in FMT studies. However, quantifying the degree of bacterial engraftment is not standardized and the factors that promote engraftment of individual strains remain elusive.^39^ A study by Rossen *et al*. demonstrated that engraftment of donor species contributed to the success of FMT, since responders who received allogenic faeces had greater similarity to their donor’s microbiota.^12^ More recently, the results of the comprehensive meta-analysis by Ianiro *et al*. suggests that higher donor strain engraftment is associated with improved clinical outcomes across a variety of clinical indications but not per indication,^40^ whereas the results of another comprehensive meta-analysis did not determine an association.^41^ Podlesney *et al*. found that the role of engraftment and clinical outcomes is probably disease-dependent and suggested that IBD patients rather benefit from targeted FMT strategies with the goal to induce engraftment of specific donor species or strains instead of maximizing overall engraftment.^37^ Each meta-analysis reassessed engraftment from metagenomes collected in clinical trials in various disease indications, specifically analysing sample ‘triads’ consisting of donor and pre- and post-FMT samples from recipients. This highlights how, even when using strain-level tracking, there is still considerable heterogeneity in outcomes and dependence of strain tracking and modelling methodologies.^42^ Additionally, interpretations of results may be hampered due to considerable variability in study methodologies of FMT trials in IBD, including number of individual donors and their relationship to the patient, FMT delivery method, frequency of and time intervals between FMT treatments, use of antibiotic pretreatments, and differences in primary endpoint definitions.^10^ To study engraftment in our relatively small randomized controlled trial, we used three different metrics to provide insights into the displacement of recipient microbiota by donor, the diversity of donor-derived species engrafting, and the recipient’s exposure to donor strains during and after FMT treatment. By combining these three measures we represent different facets of engraftment in one comprehensive metric. All outcomes in the total engraftment score are similar to those observed in the separate analyses, demonstrating that in the current study population, total engraftment is not predictive of reaching a state of remission in UC regardless of the calculation method. Although we think our methods take into account the major concepts of engraftment processes and provide a robust result, we cannot exclude the possibility that other engraftment metrics may correlate better with UC remission. In our pilot study, we did not find a significantly higher total engraftment in patients with a good clinical response after FMT and pretreatment with budesonide did not affect engraftment. More interestingly, we found lower total engraftment in the patients treated by a donor who was associated with better clinical outcomes. This suggests that overall bacterial engraftment may not be the sole indicator for clinical success, but that specific bacteria may be involved in the observed clinical effects of FMT in patients with UC.^28^ However, the sample size of this study as well as the resolution of taxonomic assignment are notable limitations. For instance, strain-level tracking of donor and recipient microbiomes may facilitate greater differentiation between recipient- and donor-originating taxa.^31^

Taxonomic associations were further investigated with RFC modelling and we found that the abundance of several genera belonging to Clostridia clusters IV and XIVa were most predictive of response, which is consistent with previous studies.^12,43^ Health-associated Clostridia, and their produced metabolites, appear to promote several innate and adaptive anti-inflammatory phenotypes *in vivo*^44^ and are associated with reduced inflammatory responses in humans.^45^ Remarkably, our analysis also identified that a high abundance of *Dialister* immediately following FMT was predictive of non-response. Interestingly, this genus was found at higher abundance at baseline in the non-responder individuals, which agrees with multiple studies observing that higher abundance of *Dialister* correlates with inflammation or with failed response to anti-IBD therapies in humans.^46^ However, models that relied on baseline microbiome composition alone were not predictive of response. Overall, these associations could guide a first step towards further research into targeting key [isolated] bacteria and donor–patient matching, which may ultimately result in a personalized FMT treatment approach.

Limitations apply to the present study. First, the number of patients was relatively small and the difference in clinical response between the two rationally selected donors led to an unbalanced dataset and hindered the comparison between treatment arms. Also, patients randomized to budesonide pretreatment had significantly more often been treated with anti-TNF-α therapy in the past, which suggests that those patients had a more complicated course of UC prior to study participation. This precludes a definite conclusion regarding the presumed beneficial effects of anti-inflammatory treatment prior to FMT. Furthermore, 3 weeks of budesonide 9 mg has limited anti-inflammatory potential, and may not suffice to show a difference in engraftment between treatment groups. Therefore, an effect of pretreatment or induction therapy on FMT donor engraftment cannot convincingly be excluded. Finally, a standard method to assess engraftment still remains to be determined, which means that conclusions should be interpreted considering limitations of the applied method to assess engraftment. Still, although this was a pilot study with a small group of patients, this study provides new insights into the use of FMT for IBD and may help the design of future FMT trials for patients with UC.

In conclusion, in our small randomized study, pretreatment with 3 weeks of budesonide did not significantly influence engraftment of donor microbiota and engraftment was not associated with clinical outcome. However, a significant donor-dependent effect of FMT in UC was seen, where caution is advised when interpretating this results, with the small number of patients included. Interestingly, patients treated with FMT suspensions from the donor who was associated with clinical response had lower engraftment, suggesting that response might be related to specific microorganisms instead of overall engraftment. Future studies must address the optimal selection of both patients and donors, the timing of FMT, and the combination of FMT with certain immunosuppressants.

## Supplementary Data

Supplementary data are available online at *ECCO-JCC* online.

jjae043_suppl_Supplementary_Data

## Data Availability

The data that support the findings of this study are available from the corresponding author, upon reasonable request.

## Notes

The study was registered in the Netherlands Trial Register, with reference number NL9858.

## References

[1] Chang JT. Pathophysiology of inflammatory bowel diseases. N Engl J Med 2020;383:2652–64. 33382932 10.1056/NEJMra2002697

[2] Harbord M , Eliakim R, Bettenworth D, et al; European Crohn’s and Colitis Organisation [ECCO]. Third European evidence-based consensus on diagnosis and management of ulcerative colitis. part 2: current management. J Crohns Colitis 2017;11:769–84. 28513805 10.1093/ecco-jcc/jjx009

[3] Parragi L , Fournier N, Zeitz J, et al; Swiss IBD Cohort Study Group. Colectomy rates in ulcerative colitis are low and decreasing: 10-year follow-up data from the Swiss IBD Cohort Study. J Crohns Colitis 2018;12:811–8. 29617750 10.1093/ecco-jcc/jjy040

[4] Eastaff-Leung N , Mabarrack N, Barbour A, Cummins A, Barry S. Foxp3+ regulatory T cells, Th17 effector cells, and cytokine environment in inflammatory bowel disease. J Clin Immunol 2010;30:80–9. 19936899 10.1007/s10875-009-9345-1

[5] Alam MT , Amos GCA, Murphy ARJ, Murch S, Wellington EMH, Arasaradnam RP. Microbial imbalance in inflammatory bowel disease patients at different taxonomic levels. Gut Pathog 2020;12:1–8. 31911822 10.1186/s13099-019-0341-6 PMC6942256

[6] Zhang SL , Wang S-N, Miao C-Y. Influence of microbiota on intestinal immune system in ulcerative colitis and its intervention. Front Immunol 2017;8:1–11. 29234327 10.3389/fimmu.2017.01674 PMC5712343

[7] Rinninella E , Raoul P, Cintoni M, et al. What is the healthy gut microbiota composition? A changing ecosystem across age, environment, diet, and diseases. Microorganisms 2019;7:14. 30634578 10.3390/microorganisms7010014 PMC6351938

[8] Venegas DP , De la Fuente MK, Landskron G, et al. Short chain fatty acids (SCFAs)-Mediated gut epithelial and immune regulation and its relevance for inflammatory bowel diseases. Front Immunol 2019;10:277 30915065 10.3389/fimmu.2019.00277 PMC6421268

[9] van Nood E , Vrieze A, Nieuwdorp M, et al. Duodenal infusion of donor feces for recurrent *Clostridium difficile*. N Engl J Med 2013;368:407–15. 23323867 10.1056/NEJMoa1205037

[10] Paramsothy S , Paramsothy R, Rubin DT, et al. Faecal microbiota transplantation for inflammatory bowel disease: a systematic review and meta-analysis. J Crohns Colitis 2017;11:S380–S1. 10.1093/ecco-jcc/jjx063 28486648

[11] Moayyedi P , Surette MG, Kim PT, et al. Fecal microbiota transplantation induces remission in patients with active ulcerative colitis in a randomized controlled trial. Gastroenterology 2015;149:102–9.e6. 25857665 10.1053/j.gastro.2015.04.001

[12] Rossen NG , Fuentes S, van der Spek MJ, et al. Findings from a randomized controlled trial of fecal transplantation for patients with ulcerative colitis. Gastroenterology 2015;149:110–8.e4. 25836986 10.1053/j.gastro.2015.03.045

[13] James D Lewis SC , Lisa N, Gar RL, Faten NA, Jonas HF. Use of the non-invasive components of the Mayo score to assess clinical response in ulcerative colitis. Inflamm Bowel Dis 2008;1660:6. 10.1002/ibd.20520 PMC2597552 18623174

[14] Julious SA. Sample size of 12 per group rule of thumb for a pilot study. Pharm Stat 2005;4:287–91.

[15] Terveer EM , van Beurden YH, Goorhuis A, et al. How to: establish and run a stool bank. Clin Microbiol Infect 2017;23:924–30. 28529025 10.1016/j.cmi.2017.05.015

[16] van Lingen E. TEM , van der Meulen-de Jong AE, Vendrik KEW, et al. Advances in stoolbanking. Microb Health Dis 2020;1:1–8.

[17] Keller JJ , Ooijevaar RE, Hvas CL, et al. A standardised model for stool banking for faecal microbiota transplantation: a consensus report from a multidisciplinary UEG working group. United Eur Gastroent 2021;9:229–47. 10.1177/2050640620967898 PMC8259288 33151137

[18] Elinav E , Strowig T, Kau AL, et al. NLRP6 inflammasome regulates colonic microbial ecology and risk for colitis. Cell 2011;145:745–57. 21565393 10.1016/j.cell.2011.04.022 PMC3140910

[19] Iljazovic A , Roy U, Gálvez EJC, et al. Perturbation of the gut microbiome by *Prevotella* spp. enhances host susceptibility to mucosal inflammation. Mucosal Immunol. 2021;14:113–24. 32433514 10.1038/s41385-020-0296-4 PMC7790746

[20] Louis P , Flint HJ. Formation of propionate and butyrate by the human colonic microbiota. Environ Microbiol 2017;19:29–41. 27928878 10.1111/1462-2920.13589

[21] Baldelli V , Scaldaferri F, Putignani L, Del Chierico F. The role of Enterobacteriaceae in gut microbiota dysbiosis in inflammatory bowel diseases. Microorganisms 2021;9:697. 33801755 10.3390/microorganisms9040697 PMC8066304

[22] Bian XY , Wu W, Yang L, et al. Administration of *Akkermansia muciniphila* ameliorates dextran sulfate sodium-induced ulcerative colitis in mice. Front Microbiol 2019;10:2259. 31632373 10.3389/fmicb.2019.02259 PMC6779789

[23] Khor B , Gardet A, Xavier RJ. Genetics and pathogenesis of inflammatory bowel disease. *Nature* 2011;474:307–17. 21677747 10.1038/nature10209 PMC3204665

[24] Hinz A , Kohlmann T, Stöbel-Richter Y, Zenger M, Brähler E. The quality of life questionnaire EQ-5D-5L: psychometric properties and normative values for the general German population. Qual Life Res 2014;23:443–7. 23921597 10.1007/s11136-013-0498-2

[25] Dsouza M , Menon R, Crossette E, et al. Colonization of the live biotherapeutic product VE303 and modulation of the microbiota and metabolites in healthy volunteers. Cell Host Microbe 2022;30:583–98.e8. 35421353 10.1016/j.chom.2022.03.016

[26] Ott SJ , Waetzig GH, Rehman A, et al. Efficacy of sterile fecal filtrate transfer for treating patients with clostridium difficile infection. Gastroenterology 2017;152:799–811.e7. 27866880 10.1053/j.gastro.2016.11.010

[27] Tamburini FB , Andermann TM, Tkachenko E, Senchyna F, Banaei N, Bhatt AS. Precision identification of diverse bloodstream pathogens in the gut microbiome. Nat Med 2018;24:1809–14. 30323331 10.1038/s41591-018-0202-8 PMC6289251

[28] Paramsothy S , Nielsen S, Kamm MA, et al. Specific Bacteria and metabolites associated with response to fecal microbiota transplantation in patients with ulcerative colitis. Gastroenterology 2019;156:1440–54.e2. 30529583 10.1053/j.gastro.2018.12.001

[29] Vermeire S , Joossens M, Verbeke K, et al. Donor species richness determines faecal microbiota transplantation success in inflammatory bowel disease. J Crohns Colitis 2016;10:387–94. 26519463 10.1093/ecco-jcc/jjv203 PMC4946755

[30] Kump P , Wurm P, Gröchenig HP, et al. The taxonomic composition of the donor intestinal microbiota is a major factor influencing the efficacy of faecal microbiota transplantation in therapy refractory ulcerative colitis. Aliment Pharmacol Ther 2018;47:67–77. 29052237 10.1111/apt.14387 PMC5765501

[31] Smith BJ , Piceno Y, Zydek M, et al. Strain-resolved analysis in a randomized trial of antibiotic pretreatment and maintenance dose delivery mode with fecal microbiota transplant for ulcerative colitis. Sci Rep 2022;12:5517. 35365713 10.1038/s41598-022-09307-5 PMC8976058

[32] Haran JP , Bhattarai SK, Foley SE, et al. Alzheimer’s disease microbiome is associated with dysregulation of the anti-inflammatory P-Glycoprotein pathway. Mbio 2019;10:e00632–19. 31064831 10.1128/mBio.00632-19 PMC6509190

[33] Kursa MB , Rudnicki W. Feature selection with the boruta package. J Stat Softw 2010;36:1–13.

[34] Altmann A , Toloşi L, Sander O, Lengauer T. Permutation importance: a corrected feature importance measure. Bioinformatics 2010;26:1340–7. 20385727 10.1093/bioinformatics/btq134

[35] Clément Bénard GB , da Veiga S, Scornet S. Interpretable Random Forests via Rule Extraction 2020. hal-02557113v1.

[36] Wilson BC , et al. The super-donor phenomenon in fecal microbiota transplantation. Front Cell Infect Mi. 2019;9:2. 10.3389/fcimb.2019.00002 PMC6348388 30719428

[37] Podlesny D , Durdevic M, Paramsothy S, et al. Identification of clinical and ecological determinants of strain engraftment after fecal microbiota transplantation using metagenomics. Cell Rep Med 2022;3:100711. 35931074 10.1016/j.xcrm.2022.100711 PMC9418803

[38] Smillie CS , Sauk J, Gevers D, et al. Strain tracking reveals the determinants of bacterial engraftment in the human gut following fecal microbiota transplantation. Cell Host Microbe 2018;23:229–40.e5. 29447696 10.1016/j.chom.2018.01.003 PMC8318347

[39] Toit A. Principles of microbiota engraftment. Nat Rev Microbiol 2018;16:186. 10.1038/nrmicro.2018.29 29479073

[40] Ianiro G , Punčochář M, Karcher N, et al. Variability of strain engraftment and predictability of microbiome composition after fecal microbiota transplantation across different diseases. Nat Med 2022;28:1913–23. 36109637 10.1038/s41591-022-01964-3 PMC9499858

[41] Schmidt TSB , Li SS, Maistrenko OM, et al. Drivers and determinants of strain dynamics following fecal microbiota transplantation. Nat Med 2022;28:1902–12. 36109636 10.1038/s41591-022-01913-0 PMC9499871

[42] Lavelle A , Sokol H. Understanding and predicting the efficacy of FMT. Nat Med 2022;28:1759–60. 36109641 10.1038/s41591-022-01991-0

[43] Nishida A , Inoue R, Inatomi O, Bamba S, Naito Y, Andoh A. Gut microbiota in the pathogenesis of inflammatory bowel disease. Clin J Gastroenterol 2018;11:1–10. 29285689 10.1007/s12328-017-0813-5

[44] Atarashi K , Tanoue T, Oshima K, et al. T-reg induction by a rationally selected mixture of Clostridia strains from the human microbiota. Nature 2013;500:232–6. 23842501 10.1038/nature12331

[45] Olendzki B , Bucci V, Cawley C, et al. Dietary manipulation of the gut microbiome in inflammatory bowel disease patients: Pilot study. Gut Microbes 2022;14:2046244. 35311458 10.1080/19490976.2022.2046244 PMC8942410

[46] Ventin-Holmberg R , et al. The gut fungal and bacterial microbiota in pediatric patients with inflammatory bowel disease introduced to treatment with anti-tumor necrosis factor-alpha. Sci Rep 2022;12:6654. 35459927 10.1038/s41598-022-10548-7 PMC9033777

