# Supplementary material for: Faecal Microbiota Transplantation Engraftment After Budesonide or Placebo in Patients With Active Ulcerative Colitis Using Pre-selected Donors: A Randomized Pilot Study
Source: J Crohns Colitis. 2024 Apr 4;18(9):1381–93. doi: 10.1093/ecco-jcc/jjae043 (PMC11369067; doi:10.1093/ecco-jcc/jjae043)
Supplement: jjae043_suppl_Supplementary_Data [file jjae043_suppl_supplementary_data.docx]

**Supplementary results**

*Taxonomic associations with FMT response*

To determine the best performing model, we ran RFC models at different taxonomic levels. We found that the model accounting for all time points and with abundances aggregated at the genus level achieves the highest performance both in terms of AUC and F1 score using a Leave-One-Out Cross-Validation (LOOCV) approach (Supplemental Fig. 5A). We chose this model for the remaining analysis and ran Permutated Importance calculations on the RFC output to identify Genera whose abundance is highly predictive of response (Supplemental Fig. 5B, 5C). Results from RFC as from all other machine learning modeling approaches are difficult to interpret. Several post-hoc methods have been recently developed to aid human interpretability to the predictions obtained from RF analysis. We therefore applied the Stable and Interpretable RUle Set (SIRUS) pipeline to the output of the best predictive RFC model(1). SIRIUS identified nine highly predictive logical rules of the form “If the abundance of Genus X at time T is higher than C, then the probability of response is K%” (Supplementary figure 6).

*Quality of life*

In the responder group (n=10), the median total score of the EQ-5D-5L was 57.5 (31.0-81.3) at baseline, which was increased to 85.0 (75-90) points 4 weeks after FMT (p<0.04) and 84.5 points (71.3-91.2) 8 weeks after FMT (p<0.01). Unfortunately, we could not assess changes in quality of life in non-responders, as some of them did not complete study follow up due to progressive symptoms.


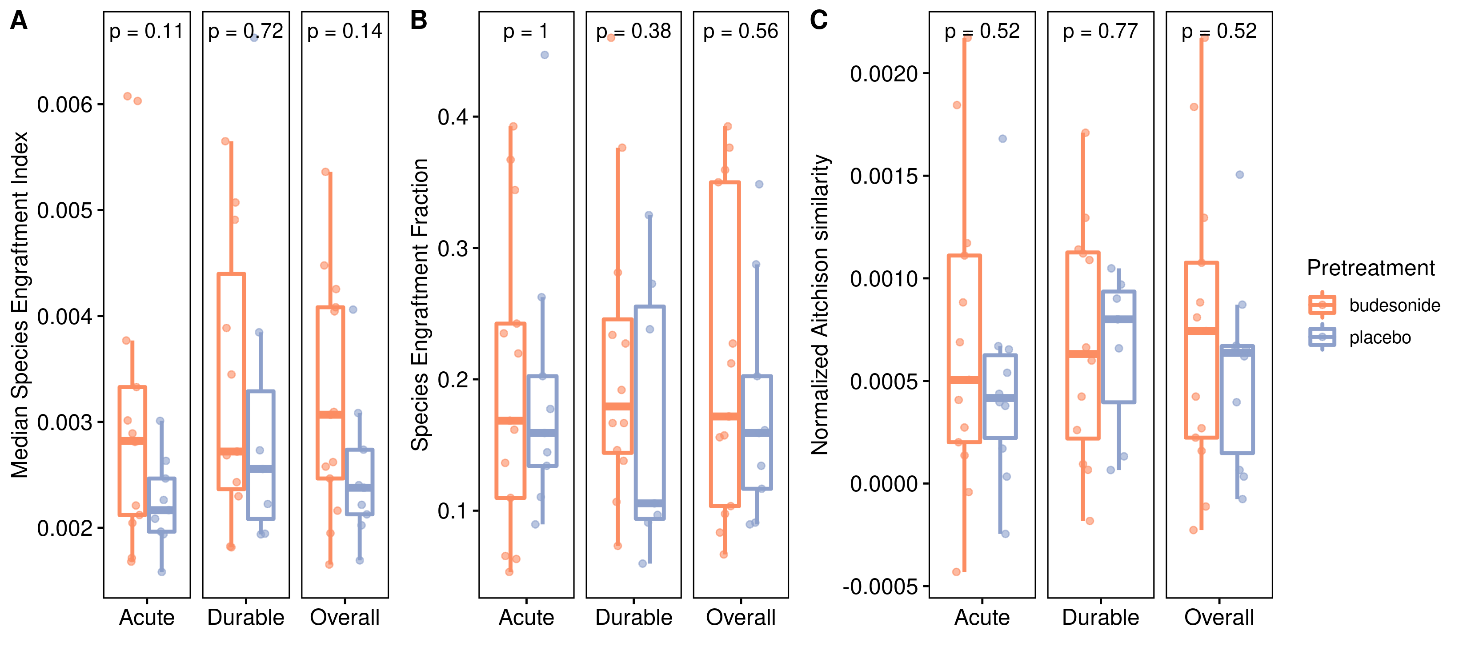
**Supplementary Figure 1. Median Species Engraftment Index (SEI), Species Engraftment Fraction (SEF) and Aitchison similarity to donor scores compared between budesonide and placebo-treated patients.** A) The median SEI, B) SEF and 3) Aitchison similarity are not significantly altered by budesonide pretreatment (P > 0.05).
Each panel shows the P-values of Wilcoxon rank sum exact tests between the two differently colored groups.
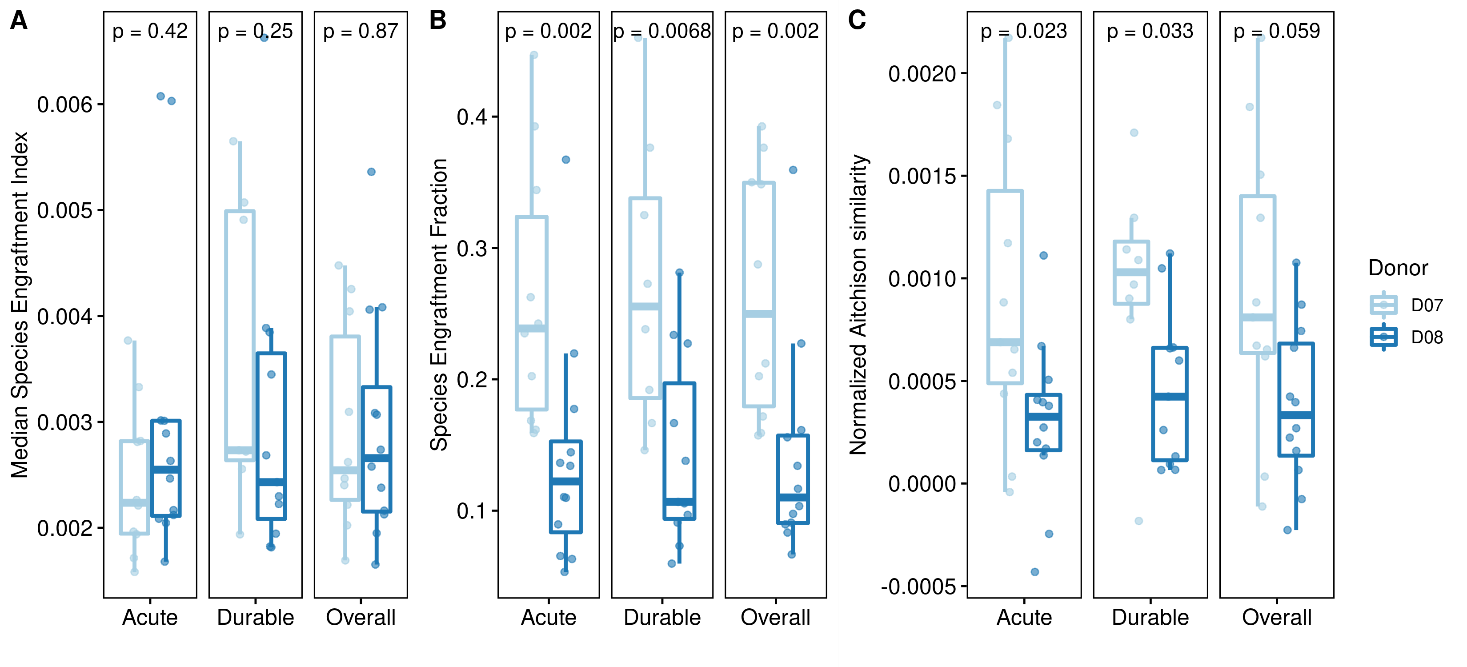
**Supplementary Figure 2. Total Species Engraftment Index, Species Engraftment Fraction and Aitchison similarity to donor scores compared between patients treated with microbiota from D07 and D08.** A) Median SEI is not different between patients treated with FMT from donor D07 and D08 (P > 0.05). B) SEF (all timeframes) and C) normalized Aitchison distances (acute and durable, but not overall) are increased in patients that received FMT from donor D07 (P < 0.05; Wilcoxon rank sum exact test).


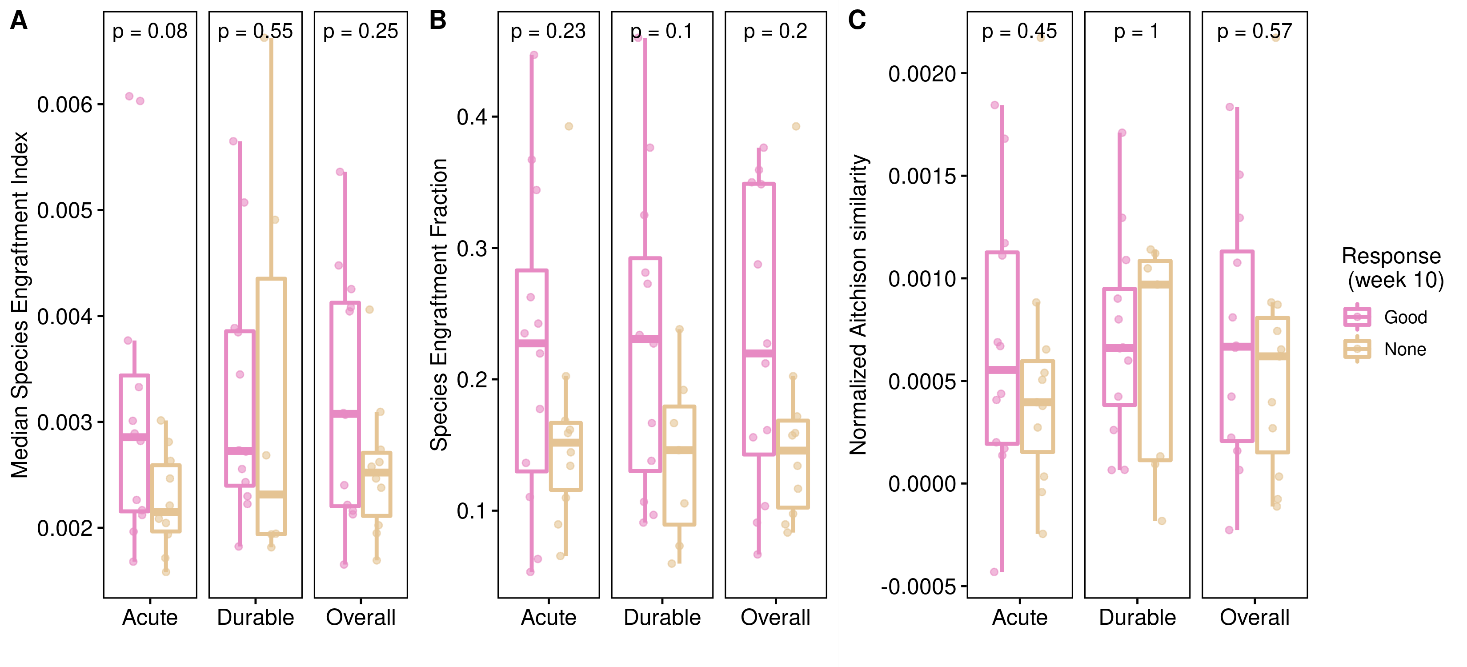


**Supplementary Figure 3. Total Species Engraftment Index, Species Engraftment Fraction and Aitchison similarity to donor scores compared between patients with a good clinical response and those with no positive clinical outcome at week 10 (4 weeks after FMT).** A) Median SEI, B) number of engrafted species (SEF) or C) the similarity to the donor’s bacterial composition (Aitchison similarity) was not different between patients with a good or bad clinical response (P > 0.05; Wilcoxon rank sum exact test).


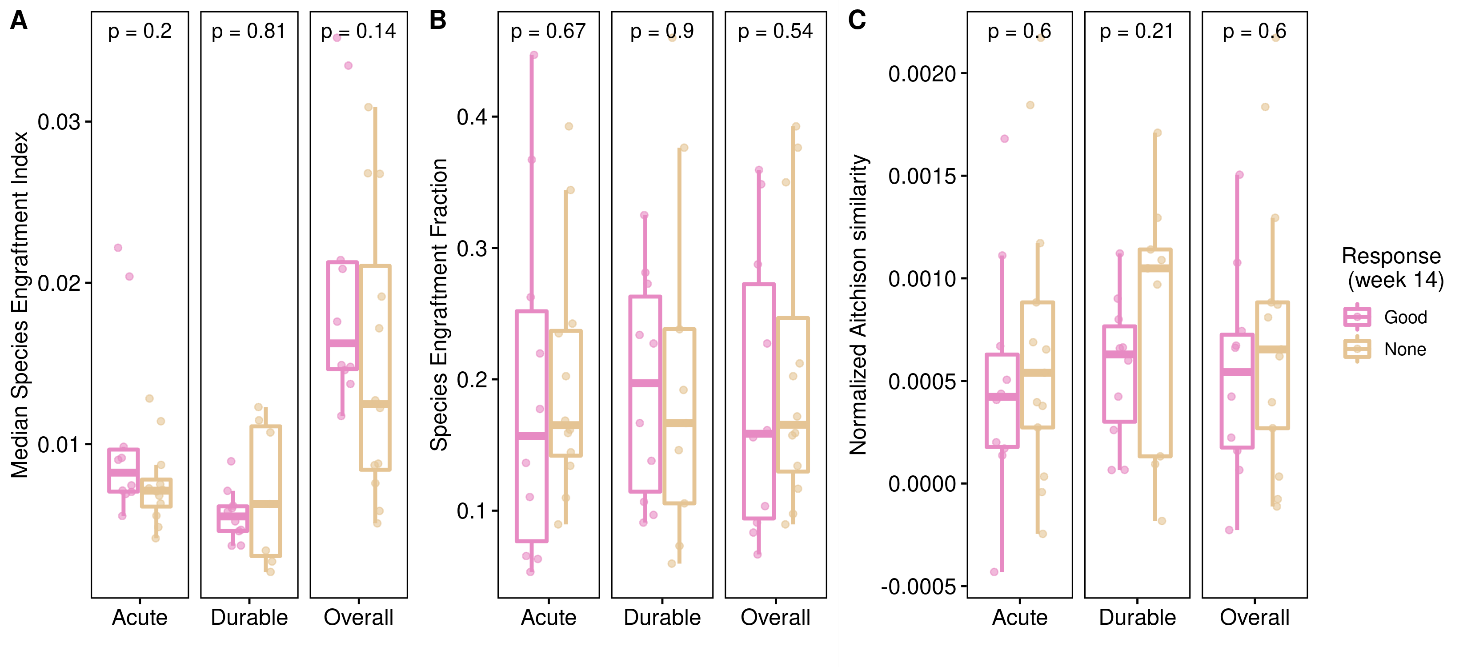
**Supplementary Figure 4. Total Species Engraftment Index, Species Engraftment Fraction and Aitchison similarity to donor scores compared between patients with a good clinical response and those with no positive clinical outcome at week 14 (8 weeks after FMT).** At week 14, there is no difference in engraftment between responders and non-responders by any metric (P > 0.1; Wilcoxon rank sum exact test).


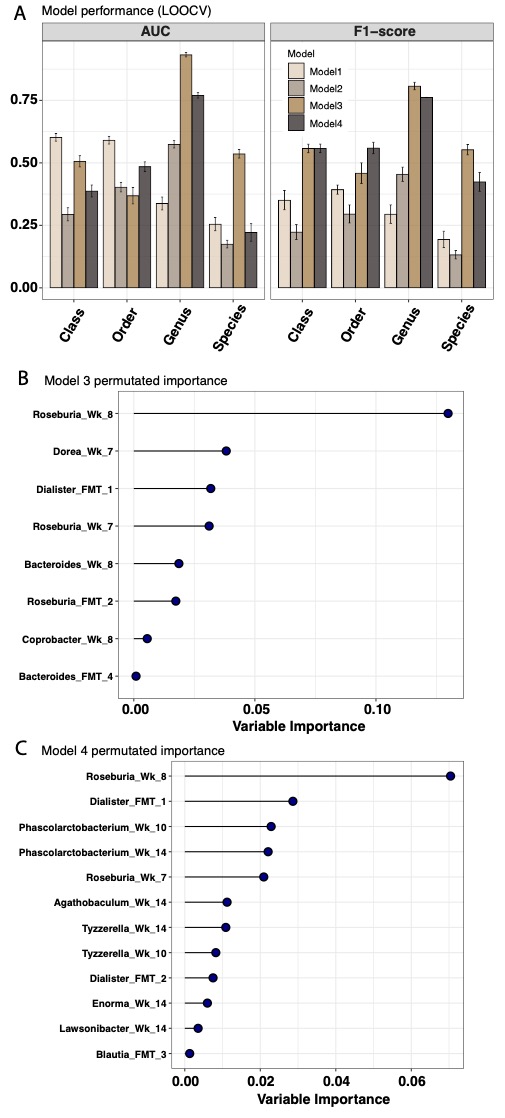


**Supplementary Figure 5:** A) Classification performance estimated as Area Under the Curve and F1-score (harmonic mean between precision and recall) was determined for different RFC models corresponding to different combinations between time points and microbiome abundance aggregated at different taxonomic levels. The following models were used: baseline samples only (model 1); baseline and post FMT samples (model 2); baseline, post FMT and week 7&8 (model 3); all timepoints up to week 14 (model 4).

B) Permutated variable importance for the most predictive model (Model 3 with Genus level aggregation), identifies as most predictive of outcome the abundance of known health-associated bacteria (e.g. *Roseburia*, and *Dorea*) post FMT. Model also finds as highly predictive the abundance of Genus *Dialister* right after FMT.

C) Similar analysis is displayed for Model 4 with abundance aggregated at the Genus level. Compared to Model 3 we observe that abundance at later time points of the Clostridia genus *Phascolarctobacterium* to be strong contributor to predicting outcome.


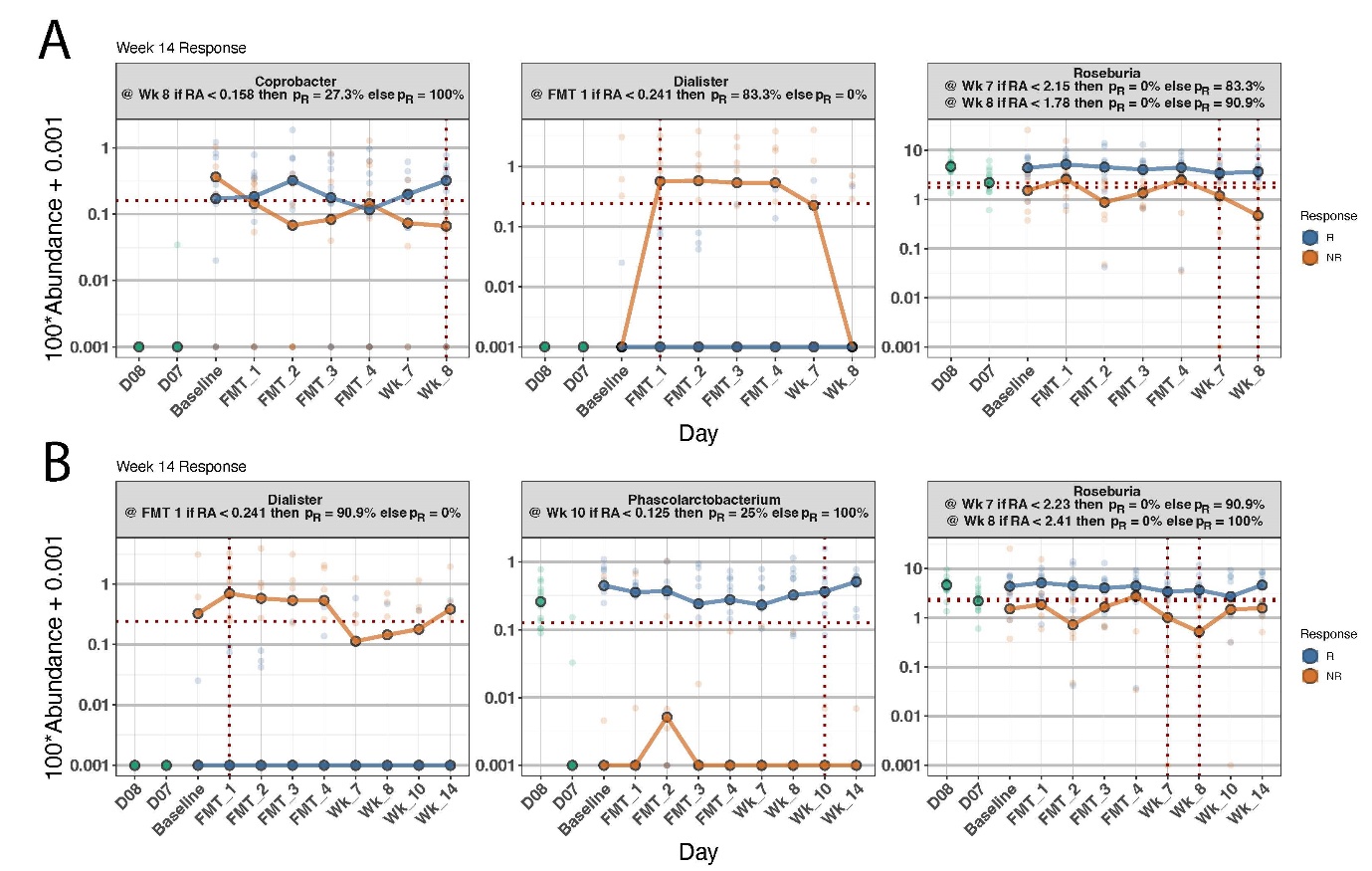


**Supplementary Figure 6. Taxonomic associations with FMT response**

Relative abundances of genera in longitudinal samples of recipients that are most predictive in discriminating between responders (blue points & solid lines) and non-responders (orange points & solid lines) determined from RFC model. The solid lines represent the median relative abundances in responders and non-responder groups with relative abundances from individuals as lighter points. The red dashed lines in each panel highlight the most discriminative time point and the model identified threshold determined by Model 3 consisting of Baseline + FMT + Week-7 + Week-8 samples only (panel A) and Model 4 consisting of all timepoints up to Week 14 (panel B).

**References supplementary**

1. Benard C, Biau G, Da Veiga S, Scornet E. SIRUS: Stable and Interpretable RUle Set for classification. Electron J Stat. 2021;15(1):427-505.
